# Can elastography predict early allograft dysfunction or loss after liver transplantation? A prospective study of diagnostic accuracy

**DOI:** 10.1016/j.clinsp.2025.100634

**Published:** 2025-05-01

**Authors:** Guilherme Marques Andrade, Luiz Marcelo Sá Malbouisson, Denise Paranaguá Vezozzo, Wellington Andraus, Paula Sepulveda Mesquita, Luiz Augusto Carneiro D'Albuquerque, Alberto Queiroz Farias, Flair José Carrilho

**Affiliations:** a Department of Gastroenterology, Faculdade de Medicina da Universidade de São Paulo (FMUSP), São Paulo, SP, Brazil; b Division of Anesthesiology, Hospital das Clínicas da Faculdade de Medicina da Universidade de São Paulo (HCFMUSP), São Paulo, SP, Brazil

**Keywords:** Liver transplantation, Graft dysfunction, Early allograft dysfunction, Allograft loss, Liver elastography

## Abstract

**Introduction:**

The imbalance between the demand for liver transplants and the shortage of donors can be addressed by expanding the donor pool, including using extended criteria donors. This strategy may reduce waiting time and list mortality but can increase poor graft function rates, affecting short-term outcomes. Tools to predict and diagnose Early Allograft Dysfunction (EAD) are crucial. Elastography for Liver Stiffness Measurement (LSM) may predict EAD and graft loss early post-transplant.

**Methods:**

In this prospective observational study, the authors assessed the diagnostic accuracy of elastography for predicting EAD or loss in liver transplant recipients admitted to the ICU of Hospital das Clínicas, Universidade de São Paulo, from 2016 to 2018. Patients underwent daily LSM from ICU admission to day 7 post-transplant. EAD was defined by Olthoff et al.'s criteria, and allograft loss was defined by the need for retransplantation or death within 180 days.

**Results:**

EAD developed in 27 patients (44.3 %). The median LSM was 2.12 m/s (IQR 1.87–2.67 m/s) for the EAD group and 1.70 m/s (IQR 1.55–1.90 m/s) for the non-EAD group. For predicting EAD, elastography on day 1 had a c-statistic of 0.83, sensitivity 41 %, specificity 97 %, and accuracy 83 % at a cutoff of 2.39 m/s. For predicting early allograft loss, the c-statistic was 0.93, with a sensitivity 76 %, specificity 100 %, and accuracy 93 % at a cutoff of 2.25 m/s on day 1.

**Conclusion:**

Elastography demonstrated robust performance in predicting EAD and early graft loss post-transplant, outperforming traditional prognostic scores. Further multicenter studies are needed to confirm these findings.

## Highlights

• Elastography can be used to measure liver stiffness after transplantation.
• Elastography had good accuracy in predicting allograft loss.
• Elastography has the advantage of prediction dysfunction on the first day.

## Introduction

The imbalance between the demand for liver transplants and the shortage of donors could be partially addressed by expanding the donor pool, including the use of livers from extended criteria donors^1^ Although this strategy may reduce waiting time and list mortality, it can also lead to increased rates of poor graft function, impacting short-term patient and graft survival.

Significant effort has been devoted to developing tools to predict and diagnose Early Allograft Dysfunction (EAD) and loss^2^, ^3^, ^4^, ^5^, ^6^ These tools include pre-operative scoring systems, various single^7^, ^8^, ^9^, ^10^ or combined variables^11^, ^12^, ^13^, ^14^ function tests^15^ assessment of hepatocyte metabolism of 13C-labelled methacetin by the cytochrome P450 1A2 (LiMax)^16^ and the identification of early transcriptional signatures associated with initial poor graft function^17^

The combination of liver biochemistry tests proposed by Olthoff et al. is the most widely used set of criteria for EAD. These criteria include AST or ALT levels higher than 2000 IU/L within the first week and/or bilirubin ≥10 mg/dL and/or an INR ≥1.6 on day 7 after liver transplantation^12^ However, these criteria may have limited applicability for very early diagnosis, given that up to 60 % of graft losses occur within the first few days post-transplant^18^

Liver Stiffness Measurement (LSM) using elastography has been examined for liver allograft evaluation, focusing primarily on late post-transplantation periods, such as disease recurrence and fibrosis progression^19^^,^^20^ late clinical outcomes^21^^,^^22^ and acute cellular rejection^23^ However, studies on elastography in the perioperative phase of transplant are rare and primarily assess living donors' livers^24^, ^25^, ^26^, ^27^ Its applicability in the early postoperative period of liver transplantation has not been sufficiently explored.

The authors hypothesized that postoperative changes, including cell damage during procurement, allograft storage, and transplantation could increase stiffness, be detectable by elastography, and predict the development of early allograft dysfunction and loss.

## Methods

### Study design and population

In this prospective observational study of diagnostic accuracy, the authors followed the STARD guidelines (Standards for Reporting of Diagnostic Accuracy Studies)^28^ to report the development and validation of a cohort for assessing the predictive value of elastography for graft outcomes after liver transplantation. The primary objective was to assess the diagnostic accuracy of elastography to predict EAD or loss. Secondary objectives included comparing the diagnostic accuracy of elastography with the main prognostic scores.

All liver transplant recipients consecutively admitted to the Intensive Care Unit (ICU) of the Department of Gastroenterology, Hospital das Clínicas of the University of São Paulo School of Medicine, Brazil, from 2016 to 2018, were assessed for eligibility. Inclusion criteria were deceased donor adult liver transplant recipients in the immediate postoperative period, over 18 years of age, with consent to participate. Exclusion criteria included the use of a temporary open abdomen strategy and technical flaws in the elastography work-up. All patients underwent a stringent protocol that included a thorough physical examination, review of clinical charts, laboratory data, graft imaging, Doppler ultrasound measurements, and LSM. Various scores for predicting outcomes after liver transplantation were calculated, including MELD (2), d-MELD (5), DRI (3), SOFT (4), BAR, MELD Day-5 (13), and postoperative MEAF (6).

Primary nonfunction of the graft was diagnosed according to the Organ Procurement and Transplantation Network policy^29^ Acute-on-Chronic Liver Failure (ACLF) syndrome was diagnosed according to the EF-CLIF criteria^30^ The study was conducted in accordance with the principles of the institutional ethics board review. Written informed consent was obtained from patients before enrollment.

### Groups, diagnostic criteria, and final adjudicated diagnosis

Based on the evaluation of allograft function during the first seven days post-transplantation, two groups were formed: patients who developed EAD (*n* = 27) and those who did not (*n* = 34). The group classification was based on Olthoff's criteria and agreed upon by a panel consisting of an intensivist, hepatologist, and transplant surgeon, who examined patients daily. Allograft loss was defined by the need for retransplantation or death within 180 days. The cohort had a mean follow-up of 8.7 ± 5.1 months (median 12 months), which helped confirm the final diagnosis established according to current criteria^2^ All personnel involved in patient care were blind to LSM results.

### Protocol for elastography acquisition

Patients underwent daily LSM from ICU admission (day 1) up to postoperative day 7. All measurements were performed by a trained physician, blind to patient data, using the pSWE/ARFI® (point shearwave elastography – Acoustic Radiation Force Impulse – Siemens Acuson S2000, Siemens Medical Systems Co. Ltd Erlangen, Germany) with 4V1 transducers. A valid measurement required at least 10 valid readings, a success rate ≥ 60 %, and an Interquartile Range (IQR) less than 30 % of the median LSM value (IQR/LSM ≤ 0.3). Results were expressed as meters per second (m/s), with the median value of measurements considered for analysis. Factors that could influence results, such as ascites, hematomas, fluid collections, intrahepatic bile duct dilation, abdominal wall edema, and elevated BMI, were registered.

### Doppler ultrasound flow and hemodynamic measurement

Hepatic artery resistivity index in the common hepatic artery, portal, and hepatic vein flow velocity and waveform pattern were measured on day 1 by an expert radiologist within 12 hours from elastography. Central venous pressure, cardiac index, and pulmonary artery median pressure were measured using a Swan-Ganz catheter, with inferior vena cava diameter variation assessed by point-of-care bedside ultrasound. These data were obtained only on the first postoperative day, immediately before the first elastography acquisition.

### Liver biopsy

Tru-cut needle biopsies were routinely obtained approximately 2 to 3 hours after portal reperfusion intraoperatively. Samples were graded semi-quantitatively for ischemia-reperfusion injury severity into four grades^17^ Subsequent biopsies were taken during the post-transplant period at the attending physician's discretion.

### Ethical considerations

The study was performed in accordance with the principles of the Declaration of Helsinki. The protocol was approved by the institutional ethics board (number 1.455.859), and written informed consent was obtained from all participants.

### Statistical analysis

A sample size of at least 42 patients was calculated, assuming a mean frequency of 29.6 % for EAD, accepting a significance level of 5 % and a statistical power of 0.80^11^^,^^12^^,^^31^ To account for possible technical limitations with early postoperative ultrasound use, an increment of at least 30 % in the sample size was planned.

Continuous variables were presented as medians and interquartile ranges or means and standard deviations, and categorical variables as numbers and percentages. Sensitivity, specificity, accuracy, positive predictive values, negative predictive values, positive likelihood ratio, and negative likelihood ratio were calculated for LSM with cutoffs defined by ROC curves and Youden's J statistic. Elastography on day 1 was selected for calculating diagnostic performance using the highest LR+ and lowest LR- as cutoff points for predicting early allograft dysfunction and loss.

The performance of elastography in the development cohort was internally validated using the same cutoff values and a bootstrap method, with an optimism-corrected c-statistic calculated using 500 bootstrap samples. Comparisons of c-statistics were performed using a non-parametric test, with p-values adjusted for multiple comparisons by Bonferroni's method. Chi-Square, Fisher's exact test, and *t*-test were used. Correlations between LSM and continuous variables were made using Pearson's correlation test. Variables with statistical significance or clinical relevance were included in a Cox regression analysis for calculating odds ratios. The significance level was set at 0.05, with all p-values being two-tailed. Statistical analyses were performed using SPSS® (IBM SPSS 25.0 Statistics for Windows, Armonk, NY: IBM Corp.) and R Software® (The R Foundation for Statistical Computing Platform, Vienna, Austria).

## Results

### Development cohort

**Fig. 1:**
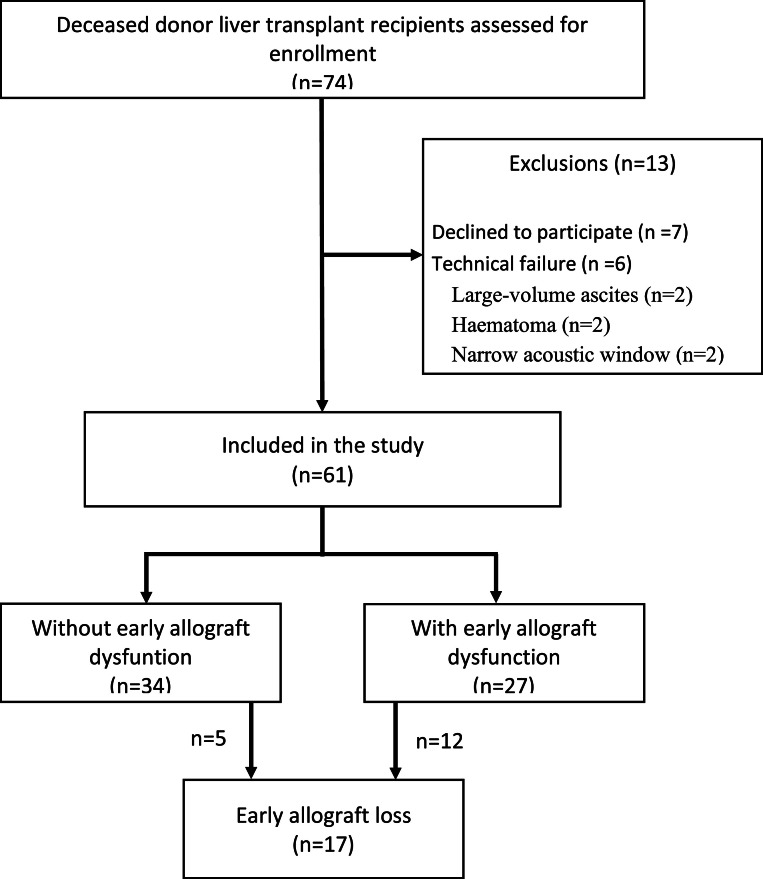
Flowchart of the study population.

During the study period, 74 liver transplant patients were screened for participation. As shown in Fig. 1, 13 were excluded, leaving 61 for analysis. The characteristics of patients, donors, grafts, and data from the transplant surgeries are shown in Table 1. Notably, the groups were comparable regarding baseline characteristics of the patients, donors, and grafts, except the group with EAD had more allografts from deceased donors with brain injury (*p* = 0.004).

**Table 1:** Characteristics of patients, donors, grafts and intraoperative data.

	Total	With early allograft dysfunction	Without early allograft dysfunction	p
	*n* = 61	*n* = 27 (44.2)	*n* = 34 (56.8)	
Patients				
Age (years)	50±14	47±14	52±14	0.22
Gender (male)	35 (57)	18 (67)	17 (49)	0.2
BMI (kg/m^2^)	26±5	26±5	26±4	0.99
Blood type				0.13
O	32 (53)	17 (63)	15 (44)	
A	22 (36)	6 (22)	16 (47)	
Etiology of liver disease				0.87
Viral	21 (38.2)	8 (33.3)	13 (41.9)	
Alcohol	6 (10.9)	4 (16.7)	2 (6.5)	
NASH	5 (9.1)	3 (12.5)	2 (6.5)	
Others	23 (41.8)	9 (37.5)	14 (45.2)	
MELD score	22±12	22±12	21.9 ± 11.5	0.96
ACLF grade 2‒3	11 (18)	5 (18.5)	6 (17.6)	0.92
Acute liver failure	3 (4.9)	1 (3.7)	2 (6)	0,6
Retransplantation	6 (9.8)	3 (11.1)	3 (8.8)	0.40
Donors				
Age (years)	36.8 ± 16.2	38.8 ± 16.8	35.1 ± 115.8	0.38
Gender (male)	38 (62.3)	16 (43)	22 (57)	0.66
BMI (kg/m^2^)	23.9 ± 4	23.9 ± 3.6	23.8 ± 4.3	0.95
ICU stay (days)	4.9 ± 3.8	5.4 ± 3.62	4.5 ± 3.9	0.37
Serum sodium (mg/dL)	155±15	157±13	153.1 ± 15.7	0.25
Cause of death (non-trauma)	35 (57.3)	14 (51.8)	21 (61.2)	0.004*
Liver grafts				
Total ischemia time (min)	492±121	504±126	482±118	0.49
Warm ischemia time (min)	33±6	33±7	32±6	0.83
Cold ischemia time (min)	459±121	471±125	450±119	0.50
Graft weight (g)	1335±332	1356±341	1317±327	0.65
Graft/body weight ratio ( %)	1.9 ± 0.6	1.9 ± 0.5	1.91±0.6	0.81
Steatosis (>30 %)	8 (13)	3 (11)	5 (15)	0.67
Ischemia-Reperfusion lesion (> grade 2)	13 (21)	8 (30)	5 (15)	0.15
Intraoperative data				
Anesthesia (min)	535±96	530±106	539±88	0.71
Surgical time (min)	412±90	410±92	413±90	0.9
Bleeding (mL)	596±524	585±457	605±578	0.88
Packed red blood cell transfusion (units)	2.1 ± 2.2	2.0 ± 2.4	2.2 ± 2.1	0.71

Results expressed as Mean ± SD or n ( %). *t*-test with Welch's correction. Chi-Square test/Fisher's exact test.

a *p* < 0.05.ACLF, Acute on Chronic Liver Failure; BMI, Body-Mass Index; ICU, Intensive Care Unit; MELD, Model for End-stage Liver Disease; NASH, Non-Alcoholic Steatohepatitis.

### Clinical follow-up

After liver transplantation, all patients were admitted to the ICU under mechanical ventilation. Mechanical ventilation was required for more than 48 hours in 51.9 % of EAD group vs. 23.5 % in the non-EAD group (*p* = 0.022). Vasopressor use was required in 66.7 % of EAD group vs. 26.5 % of non-EAD group (*p* < 0.001). ICU stay was 13.4 ± 11.9 days for the EAD group vs. 8.7 ± 0.5 days for non-EAD; *p* = 0.1. Hospital stay was 32.4 ± 25.3 days for EAD vs. 26.9 ± 21.8 days for non-EAD; *p* = 0.37. Rates of acute cellular rejection, biliary complications, and reoperations were similar between groups. Higher dialysis need was observed in EAD group (59.3 % vs. 26.5 %; *p* = 0.009). Thirteen deaths occurred after transplantation and were due to sepsis (7), multiple organ dysfunction (4), and hemorrhagic shock (2). One-year patient survival was 78.6 %, and graft survival was 71.8 %. Graft loss was 14.7 % in non-EAD vs. 44.4 % in EAD.

### Liver biopsy findings

Fifty-two patients (85 %) had at least grade I ischemia-reperfusion injury. Grade III/IV was found in 13 (21.3 %), but none of these patients developed early allograft dysfunction or loss. LSM was not significantly different between patients with ischemia-reperfusion injury grades 0/I/II or III/IV (1.92 ± 0.49 m/s vs. 1.91 ± 0.38 m/s; *p* = 0.94). The presence of steatosis on the biopsy did not correlate with LSM (*r* = 0.14; *p* = 0.275), whether stratified into mild (≤ 30 %) or severe (> 30 %) steatosis (1.75 ± 0.26 m/s vs. 1.94 ± 0.5 m/s, respectively; *p* = 0.61).

### Diagnostic performance of elastography for early allograft dysfunction

**Fig. 2:**
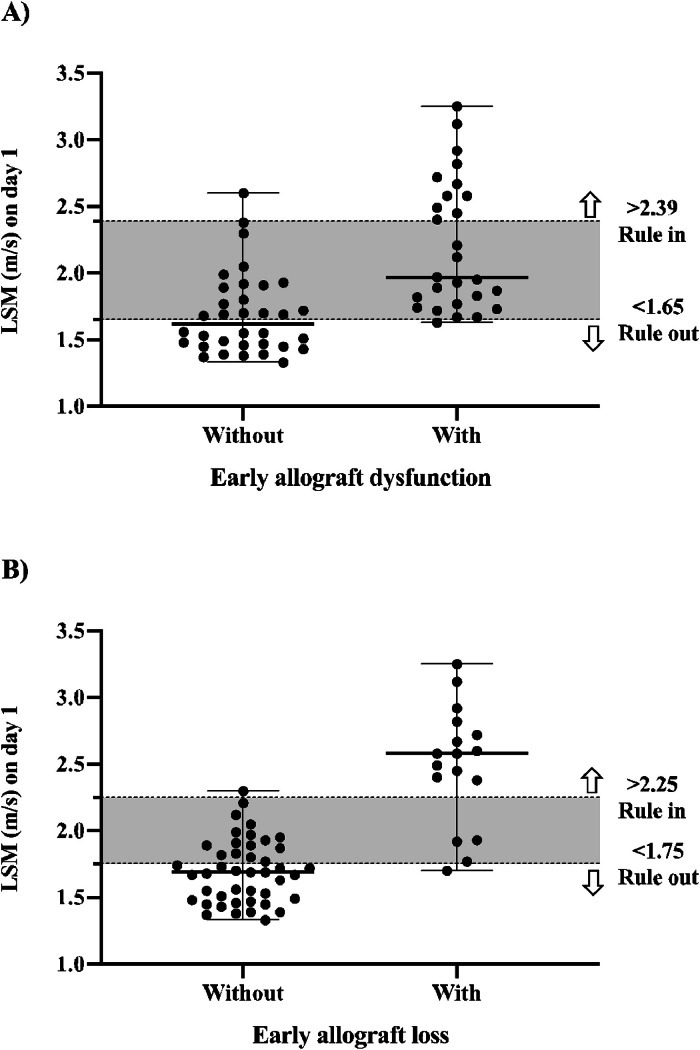
LSM cutoff points on day 1 to rule in and rule out early allograft dysfunction (A) or early allograft loss (B).

The median LSM for the whole population was 1.87 m/s (IQR 1.67–2.20 m/s), 2.12 m/s (IQR 1.87–2.67 m/s) for the group with EAD, and 1.70 m/s (IQR 1.55–1.90 m/s) for the group without EAD. As shown in Table 2, elastography performed moderately well for predicting EAD. Although the comparisons of the c-statistic on each day of LSM showed no significant difference among them. The highest absolute results were obtained on day 5, with an accuracy of 0.84, sensitivity of 0.75, specificity of 0.79, PPV of 0.72, and NPV of 0.82. Using a cutoff value of 2.39 m/s, LSM on day 1 had a sensitivity of 0.41, specificity of 0.97, PPV of 0.92, NPV of 0.67, accuracy of 0.83, LR- of 0.61, and LR+ of 13.85 to predict EAD. Using a cutoff of 1.65 m/s had a sensitivity of 0.96, specificity of 0.50, PPV of 0.60, NPV of 0.94, accuracy of 0.83, LR+ of 1.93, and LR- of 0.07 to exclude EAD, as illustrated in Fig. 2 (part A).

**Table 2:** Diagnostic performance of daily LSM after liver transplantation to predict early allograft dysfunction.

Postoperative day	LSM cutoff (m/s)	Se	Sp	PPV	NPV	AUC	LR+	LR-
Day 1	1.71	0.89	0.65	0.67	0.88	0.83 (0.73‒0.93)	2.52	0.17
Day 2	1.67	1.0	0.50	0.61	1.0	0.82 (0.72‒0.92)	2.0	0.00
Day 3	1.78	1.0	0.50	0.59	1.0	0.81 (0.73‒0.93)	2.0	0.00
Day 4	1.83	1.0	0.53	0.61	1.0	0.83 (0.73‒0.93)	2.12	0.00
Day 5	1.93	0.75	0.79	0.72	0.82	0.84 (0.74‒0.94)	3.64	0.32
Day 6	1.70	0.96	0.59	0.62	0.95	0.84 (0.73‒0.94)	2.33	0.07
Day 7	1.66	0.96	0.64	0.65	0.95	0.84 (0.74‒0.94)	2.63	0.07

AUC Area Under the receiver operating Curve; LSM, Liver Stiffness Measurements; LR+, positive Likelihood Ratio; LR-, negative Likelihood Ratio; NPV, Negative Predictive Value; PPV, Positive Predictive Value; Se, Sensitivity; Sp, Specificity.

### Diagnostic performance of elastography for early allograft loss

Elastography had a better performance for predicting early allograft loss than dysfunction. Table 3 shows the daily diagnostic performance over the first week. Overall, elastography had an accuracy ranging from 0.90 to 0.93. Peak values were observed on day 4, but without a significant increase in the accuracy rate. The comparisons of c-statistics for each day of LSM showed no significant difference between them, indicating that any day can be used to predict early allograft loss. However, LSM on day 1 performed best, with an accuracy of 0.93, sensitivity of 0.76, specificity of 1.0, PPV of 1.0, and NPV of 0.92. Using the cutoff value of 2.25 m/s, LSM on day 1 had a sensitivity of 0.76, specificity of 0.98, PPV of 0.93, NPV of 0.91, accuracy of 0.93, LR+ of 33.65, and LR- of 0.24 to predict early allograft loss. Using a cutoff of 1.75 m/s, LSM on day 1 had a sensitivity of 0.94, specificity of 0.64, PPV of 0.50, NPV of 0.97, accuracy of 0.93, LR- of 0.09, and LR+ of 2.59 to exclude early allograft loss, as shown in Fig. 2 (part B).

**Table 3:** Diagnostic performance of daily LSM after liver transplantation to predict early allograft loss.

Postoperative day	LSM cutoff (m/s)	Se	Sp	PPV	NPV	AUC	LR+	LR-
Day 1	2.34	0.76	1.00	1.00	0.92	0.93 (0.85‒1.00)	a	0.23
Day 2	2.37	0.76	0.97	0.93	0.91	0.93 (0.86‒1.00)	33.65	0.24
Day 3	2.46	0.8	0.97	0.92	0.93	0.93 (0.85‒1.00)	35.20	0.20
Day 4	2.46	0.8	0.97	0.92	0.93	0.91 (0.81‒1.00)	35.20	0.20
Day 5	2.40	0.79	0.97	0.92	0.93	0.91 (0.80‒1.00)	34.57	0.22
Day 6	2.30	0.79	0.97	0.92	0.93	0.91 (0.81‒1.00)	34.57	0.22
Day 7	2.19	0.75	0.97	0.9	0.93	0.91 (0.81‒1.00)	33	0.26

a Unmeasurable likelihood ratio because sensitivity reached 1.0.AUC, Area Under the receiver operating Curve; LSM, Liver Stiffness Measurements; LR+, positive Likelihood Ratio; LR-, negative Likelihood Ratio NPV, Negative Predictive Value; PPV, Positive Predictive Value; Se, Sensibility; Sp, Specificity.

### Validation

Internal validation of the development cohort is shown in Table 4, Table 5. For predicting EAD, LSM on day 1 yields a sensitivity of 0.88, specificity of 0.60, PPV of 0.63, NPV of 0.86, LR+ of 2.18, LR- of 0.20, and c-statistic of 0.83. For early allograft loss, LSM on day 1 had a sensitivity of 0.69, specificity of 0.95, PPV of 0.85, NPV of 0.89, LR+ of 14, and c-statistic of 0.93.

**Table 4:** Validation of the diagnostic performance of elastography for early allograft dysfunction.

Postoperative day	LSM cutoff (m/s)	Se	Sp	PPV	NPV	AUC	LR+	LR-	p
Day 1	1.71	0.88	0.60	0.63	0.86	0.83	2.18	0.20	<0.001
Day 2	1.67	0.87	0.32	0.51	0.76	0.82	1.29	0.39	<0.001
Day 3	1.78	0.90	0.35	0.50	0.82	0.81	1.38	0.29	<0.001
Day 4	1.83	0.90	0.38	0.51	0.83	0.82	1.44	0.27	<0.001
Day 5	1.93	0.63	0.74	0.63	0.74	0.84	2.39	0.50	<0.001
Day 6	1.70	0.88	0.45	0.53	0.84	0.83	1.60	0.27	<0.001
Day 7	1.66	0.91	0.57	0.60	0.90	0.84	2.13	0.15	<0.001

AUC, Area Under the receiver operating Curve; LSM, Liver Stiffness Measurements; LR+, Positive Likelihood Ratio; LR-, negative Likelihood Ratio; NPV, Negative Predictive Value; PPV, Positive Predictive Value; Se, Sensitivity; Sp, Specificity.

**Table 5:** Validation of the diagnostic performance of elastography for early allograft loss.

Postoperative day	LSM cutoff (m/s)	Se	Sp	PPV	NPV	AUC	LR+	LR-	p
Day 1	2.34	0.70	0.95	0.85	0.89	0.93	14.06	0.32	<0.001
Day 2	2.37	0.69	0.93	0.79	0.89	0.93	9.76	0.33	<0.001
Day 3	2.46	0.77	0.96	0.88	0.93	0.93	21.19	0.24	<0.001
Day 4	2.46	0.79	0.97	0.91	0.93	0.91	28.33	0.22	<0.001
Day 5	2.40	0.77	0.97	0.88	0.93	0.90	23.40	0.24	<0.001
Day 6	2.30	0.77	0.97	0.88	0.93	0.91	22.18	0.24	<0.001
Day 7	2.19	0.67	0.93	0.73	0.91	0.91	9.72	0.35	<0.001

AUC, Area Under the receiver operating Curve; LSM, Liver Stiffness Measurements; LR, positive Likelihood Ratio; LR-, negative Likelihood Ratio; NPV, Negative Predictive Value; PPV, Positive Predictive Value; Se, Sensitivity; Sp, Specificity.

### Correlation of elastography and doppler, liver hemodynamic variables, and other factors

No significant correlation was shown between LSM and fluid balance (*r* = 0.093; *p* = 0.478), bleeding (*r* = 0.032; *p* = 0.804), central venous pressure (8.5 ± 2.3 mmHg; *r* = 0.104; *p* = 0.424), cardiac index (5.6 ± 1.6 L/min/m^2; *r* = −0.077; *p* = 0.424), mean pulmonary artery pressure (20 ± 5 mmHg; *r* = 0157; *p* = 0.226), hepatic artery resistive index (0.65 ± 0.11; *r* = −0.1; *p* = 0.41), or portal velocity (67 ± 32 cm/s; *r* = 0.09; *p* = 0.48) at the 1° postoperative day. Even with stratification into patients with or without early allograft loss, there was still no significant correlation of LSM > 2.25 m/s with portal velocity (60 ± 37 cm/s vs. 67 ± 31 cm/s; *p* = 0.41), hepatic artery resistive index (0.63 ± 0.14 vs. 0.66 ± 0.11; *p* = 0.36), or central venous pressure (8 ± 2.2 vs. 8 ± 2.3 mmHg; *p* = 0.39).

LSM was not affected by the presence or absence of small ascites (1.86 ± 0.35 vs. 1.94 ± 0.52 m/s, respectively; *p* = 0.7), BMI ≥ or < 30 kg/m^2 (2.12 ± 0.57 vs. 1.86 ± 0.42 m/s, respectively; *p* = 0.30), or inferior vena cava diameter variation ≥ or < 50 % (1.95 ± 0.4 vs. 1.90 ± 0.5 m/s, respectively; *p* = 0.52).

### Predictors of early allograft dysfunction and loss

No clinical or laboratory variable was significantly associated with early graft loss: etiology of liver disease before transplantation (OR = 0.72; 95 % CI 0.15‒3.31; *p* = 0.68), ACLF (OR = 1.81; 95 % CI 0.44‒7.96; *p* = 0.39), male donor (OR = 1.13; 95 % CI 0.44‒7.96; *p* = 0.84), cold ischemia time (OR = 0.99; 95 % CI 0.99‒1.01; *p* = 0.23), graft/body weight ratio (OR = 1.12; 95 % CI 0.50‒2.50; *p* = 0.77), lactate levels (OR = 1.07; 95 % CI 0.96‒1.20; *p* = 0.21), ischemia-reperfusion injury > grade 2 (OR = 2.63; 95 % CI 0.42‒16.51; *p* = 0.30). The occurrence of EAD was the only factor significantly associated with early allograft loss (OR = 51.40; 95 % CI 1.26‒2094; *p* = 0.03).

### Comparison of elastography and prognostic scores

Table 6 shows the comparison of the diagnostic performance of LSM, lactate, prognostic scores (BAR, MELD-5, MEAF), and the occurrence of EAD and primary non-function as predictors of early allograft loss. The test that best predicted early allograft loss was LSM > 2.25 m/s on day 1 after liver transplantation, with an accuracy of 93 %. No test was as effective as LSM > 2.25 m/s on day 1, which also had the highest Odds Ratio of 12.

**Table 6:** Comparison of the diagnostic performance of different methods to predict early graft loss.

	n ( %)	OR (95 % CI)^a^	Se	Sp	PPV	NPV	AUC (95 % CI)	LR+
LSM >2.25 m/s	13 (76)	12 (4.7‒30.7)	0.76	0.98	0.93	0.91	0.93 (0.85‒1.00)	33.65
MELD-5 > 18.9	10 (71)	7.27 (2.0‒23.4)	0.71	0.74	0.47	0.88	0.76 (0.62‒0.90)	2.79
MEAF >8	9 (53)	5.06 (1.6‒7.9)	0.52	0.81	0.52	0.81	0.72 (0.57‒0.87)	2.91
Lactate ≥40 mg/dL	11 (65)	4.23 (1.3‒13.2)	0.64	0.67	0.45	0.83	0.74 (0.61‒0.87)	2.14
BAR >18	5 (29)	3.81 (1.8‒6.8)	0.29	0.97	0.83	0.78	0.66 (0.48‒0.83)	12.9
Olthoff criteria	12 (71)	3.02 (1.2‒7.5)	0.71	0.66	0.44	0.85	0.68 (0.53‒0.83)	2.07
Primary nonfunction	7 (41)	2.58 (1.2‒5.5)	0.41	0.86	0.54	0.86	0.63 (0.47‒0.80)	3.01

a All p-values were significant (< 0.05). AUC, Area Under the receiver operating Curve; LSM, Liver Stiffness Measurements; LR, Likelihood Ratio; NPV, Negative Predictive Value; OR, Odds Ratio; PPV, Positive Predictive Value; Se, Sensitivity; Sp, Specificity.

## Discussion

The use of laboratory tests and scoring systems to predict liver allograft dysfunction has been well-documented^12^, ^13^, ^14^^,^^32^ However, these methods have significant limitations, including delayed diagnosis, limited accuracy, time-consuming procedures, and the lack of a consensus on a uniform predictive tool. This study evaluated the predictive value of elastography for early allograft dysfunction or loss, focusing on daily postoperative measurements to determine the optimal cutoff value and timeframe.

The key finding is that elastography can identify patients at higher risk of early allograft dysfunction or loss and accurately rule out those at lower risk. Elastography performed on the first postoperative day showed good accuracy in predicting both early allograft dysfunction (c-statistic of 0.83) and early allograft loss (c-statistic of 0.93), with better performance for the latter. This accuracy was internally validated and sustained over the first seven postoperative days, making elastography a highly flexible tool.

For predicting early allograft loss, the current study has shown that pSWE-LSM elastography outperformed standard prediction tools such as lactate levels, MEAF, MELD-5, BAR, Olthoff criteria, and primary non-function occurrence^19^^,^^22^^,^^33^

When compared to other methods, elastography offers the advantage of early prediction on the first postoperative day. Using cutoff points based on likelihood ratios can enhance accuracy for ruling in or out early allograft dysfunction or loss. Combined with clinical judgment, elastography can help to improve the decision-making process on providing intensive care or early retransplantation.

Although the exact causes of increased liver stiffness were not determined, the study showed that post-transplant hemodynamic changes did not affect elastography's performance. In the transplant process, a combination of injuries leads to cellular hypoxia and the generation of Reactive Oxygen Species (ROS) upon reoxygenation. This sequence results in inflammation mediated by Kupffer cells and T-cells, which activate neutrophil inflammatory responses^34^ Activated neutrophils infiltrate the injured liver, accompanied by an increased expression of adhesion molecules on endothelial cells, culminating in cell death^35^^,^^36^ These changes could increase liver stiffness after transplantation, providing a mechanistic explanation for the present findings. This suggests that the method is robust despite the complex post-transplant environment, where injuries and inflammatory responses increase liver stiffness despite the absence of significant fibrosis.

The use of elastography for the detection of liver graft dysfunction after liver transplantation has advanced significantly in recent years. This non-invasive imaging technique measures liver stiffness, providing crucial insights into graft health and function^25^ Studies have shown that transient elastography and shear wave elastography are effective in identifying early signs of fibrosis and dysfunction, allowing for timely interventions^37^ These methods have become valuable tools in post-transplant monitoring, enhancing patient outcomes by enabling early detection and management of complications such as evaluation of rejection or recurrent hepatitis^38^ However, to our knowledge, there are very few studies that propose the use of hepatic elastography as a means to identify early graft dysfunction in the first days of the postoperative period after liver transplantation. In 2009, Inoue et al. demonstrated that recipients with postoperative complications had significantly higher LSM values than those without complications in the fourth postoperative week and those with acute cellular rejection had a concomitant sharp rise in liver stiffness and a rapidly depleted portal flow^23^^,^^24^ In this study, the authors propose the application of the pSWE-LSM technique as an innovative and readily available bedside tool to improve the detection of early graft disfunction.

While previous studies on elastography in liver transplantation were limited by heterogeneity, retrospective design, and delayed measurement post-transplant^19^, ^20^, ^21^, ^22^ this study's prospective approach and early measurement post-transplant enhance its predictive applicability.

However, there are some limitations to consider. The use of the pSWE-LSM technique may not be generalizable to other equipment and techniques. Additionally, this single-center study's findings should be confirmed by larger multicenter studies. Donor variables were similar between groups, though some factors could not be evaluated such as donor care, procurement, and logistics. The higher frequency of early dysfunction in this study might reflect the center's use of expanded criteria liver grafts, which are increasingly common due to the growing number of patients on liver transplantation waiting lists. Even though lower than in similar transplantation centers in the country^39^ early dysfunction was indeed more frequent than reported in the literature, thereby increasing the pre-test probability of the present sample.

In conclusion, this proof-of-concept study demonstrated that elastography within the first week after liver transplantation is a robust predictor of early allograft dysfunction or loss, with superior accuracy compared to standard scores. Integrating elastography into ICU management for transplanted patients can streamline early allograft dysfunction or loss prediction, improving patient care and survival.

## Declaration of competing interest

The authors declare no conflicts of interest.

## References

[1] Barshes N.R., Horwitz I.B., Franzini L., Vierling J.M., Goss J.A. (2007). Waitlist mortality decreases with increased use of extended criteria donor liver grafts at adult liver transplant centers. Am J Transplant.

[2] Jacob M., Copley L.P., Lewsey J.D. (2004). Pretransplant MELD score and post liver transplantation survival in the UK and Ireland. Liver Transpl.

[3] Feng S., Goodrich N.P., Bragg-Gresham J.L. (2006). Characteristics associated with liver graft failure: the concept of a donor risk index. Am J Transplant.

[4] Rana A., Hardy M.A., Halazun K.J. (2008). Survival outcomes following liver transplantation (SOFT) score: a novel method to predict patient survival following liver transplantation. Am J Transplant.

[5] Halldorson J.B., Bakthavatsalam R., Fix O., Reyes J.D., Perkins J.D. (2009). d-MELD, a simple predictor of post liver transplant mortality for optimization of donor/recipient matching. Am J Transplant.

[6] Dutkowski P., Oberkofler C.E., Slankamenac K. (2011). Are there better guidelines for allocation in liver transplantation? A novel score targeting justice and utility in the model for end-stage liver disease era. Ann Surg.

[7] Robertson F.P., Bessell P.R., Diaz-Nieto R. (2016). High serum aspartate transaminase levels on day 3 postliver transplantation correlates with graft and patient survival and would be a valid surrogate for outcome in liver transplantation clinical trials. Transpl Int.

[8] Golse N., Guglielmo N., El Metni A. (2019). Arterial lactate concentration at the end of liver transplantation is an early predictor of primary graft dysfunction. Ann Surg.

[9] Zulian M.C., Chedid M.F., Chedid A.D. (2015). Low serum factor V level: early predictor of allograft failure and death following liver transplantation. Langenbecks Arch Surg.

[10] Lesurtel M., Raptis D.A., Melloul E. (2014). Low platelet counts after liver transplantation predict early posttransplant survival: the 60-5 criterion. Liver Transpl.

[11] Gonzalez F.X., Rimola A., Grande L. (1994). Predictive factors of early postoperative graft function in human liver transplantation. Hepatology.

[12] Olthoff K.M., Kulik L., Samstein B. (2010). Validation of a current definition of early allograft dysfunction in liver transplant recipients and analysis of risk factors. Liver Transpl.

[13] Wagener G., Raffel B., Young A.T., Minhaz M., Emond J. (2013). Predicting early allograft failure and mortality after liver transplantation: the role of the postoperative model for end-stage liver disease score. Liver Transpl.

[14] Agopian V.G., Harlander-Locke M.P., Markovic D. (2018). Evaluation of early allograft function using the liver graft assessment following transplantation risk score model. JAMA Surg.

[15] Olmedilla L., Lisbona C.J., Perez-Pena J.M. (2016). Early measurement of indocyanine green clearance accurately predicts short-term outcomes after liver transplantation. Transplantation.

[16] Stockmann M., Lock J.F., Malinowski M. (2010). How to define initial poor graft function after liver transplantation? - a new functional definition by the LiMAx test. Transpl Int.

[17] Defamie V., Cursio R., Le Brigand K. (2008). Gene expression profiling of human liver transplants identifies an early transcriptional signature associated with initial poor graft function. Am J Transplant.

[18] Lucey M.R., Terrault N., Ojo L. (2013). Long-term management of the successful adult liver transplant: 2012 practice guideline by the American Association for the Study of Liver Diseases and the American Society of Transplantation. Liver Transpl.

[19] Carrion J.A., Torres F., Crespo G. (2010). Liver stiffness identifies two different patterns of fibrosis progression in patients with hepatitis C virus recurrence after liver transplantation. Hepatology.

[20] Liao C.C., Chen T.Y., Tsang L.C. (2014). The acoustic radiation force impulse elastography evaluation of liver fibrosis in posttransplantation dysfunction of living donor liver transplantation. Transplant Proc.

[21] Pfeiffenberger J., Hornuss D., Houben P. (2019). Routine liver elastography could predict actuarial survival after liver transplantation. J Gastrointestin Liver Dis.

[22] Boeken T., Lucidarme O., Mbarki E., Scatton O., Savier E., Wagner M. (2021). Association of shear-wave elastography with clinical outcomes post-liver transplantation. Clin Res Hepatol Gastroenterol.

[23] Crespo G., Castro-Narro G., Garcia-Juarez I. (2016). Usefulness of liver stiffness measurement during acute cellular rejection in liver transplantation. Liver Transpl.

[24] Inoue Y., Sugawara Y., Tamura S. (2009). Validity and feasibility of transient elastography for the transplanted liver in the peritransplantation period. Transplantation.

[25] Lee S.H., Joo D.J., Kim S.U. (2013). Graft function measured by transient elastography in living donor liver transplantation: preliminary. Transplant Proc.

[26] Ijichi H., Shirabe K., Matsumoto Y. (2014). Evaluation of graft stiffness using acoustic radiation force impulse imaging after living donor liver transplantation. Clin Transplant.

[27] Bayramov N., Yilmaz S., Salahova S. (2019). Liver graft and spleen elastography after living Liver transplantation: our first results. Transplant Proc.

[28] Bossuyt P.M., Reitsma J.B., Bruns D.E. (2015). STARD 2015: an updated list of essential items for reporting diagnostic accuracy studies. BMJ.

[29] Farmer D.G., Abu-Elmagd K. (2022). The liver and intestinal allocation policy: decades of disparity calling for action. Am J Transplant.

[30] Moreau R., Jalan R., Gines P. (2013). Acute-on-chronic liver failure is a distinct syndrome that develops in patients with acute decompensation of cirrhosis. Gastroenterology.

[31] Hoyer D.P., Paul A., Gallinat A. (2015). Donor information based prediction of early allograft dysfunction and outcome in liver transplantation. Liver Int.

[32] Pareja E., Cortes M., Hervas D. (2015). A score model for the continuous grading of early allograft dysfunction severity. Liver Transpl.

[33] Navin P.J., Olson M.C., Knudsen J.M., Venkatesh S.K. (2021). Elastography in the evaluation of liver allograft. Abdom Radiol (NY).

[34] Burne-Taney M.J., Yokota-Ikeda N., Rabb H. (2005). Effects of combined T- and B-cell deficiency on murine ischemia reperfusion injury. Am J Transplant.

[35] Liu Y., Ji H., Zhang Y. (2015). Recipient T cell TIM-3 and hepatocyte galectin-9 signalling protects mouse liver transplants against ischemia-reperfusion injury. J Hepatol.

[36] Tang Q., Dong C., Sun Q. (2022). Immune response associated with ischemia and reperfusion injury during organ transplantation. Inflamm Res.

[37] Korda D., Lenard Z.M., Gerlei Z. (2018). Shear-wave elastography for the assessment of liver fibrosis in liver transplant recipients treated for hepatitis C virus recurrence. Eur J Gastroenterol Hepatol.

[38] Yoon J.H., Lee J.Y., Woo H.S. (2013). Shear wave elastography in the evaluation of rejection or recurrent hepatitis after liver transplantation. Eur Radiol.

[39] Salvalaggio P., Afonso R.C., Felga G., Ferraz-Neto B.H. (2013). A proposal to grade the severity of early allograft dysfunction after liver transplantation. Einstein (Sao Paulo).

